# A Novel Digital Platform to Support Child and Family Mental Health in Australia (Child and Family eHub): Protocol for a Mixed Methods Evaluation

**DOI:** 10.2196/72548

**Published:** 2025-11-13

**Authors:** Suzy Honisett, Raghu Lingam, Valsamma Eapen, Brian Oldenburg, Naomi Hackworth, Harriet Hiscock, George Charalambous, Lisa Minton, Glenn Pringle, Sue Woolfenden, Kim Dalziel, John Eastwood, Sharon Goldfeld

**Affiliations:** 1 Policy and Equity Centre for Community Child Health Murdoch Children's Research Institute Melbourne Australia; 2 School of Clinical Medicine University of New South Wales Sydney Australia; 3 Academic Unit of Child Psychiatry South West Sydney Local Health District Sydney Australia; 4 Ingham Institute Sydney Australia; 5 Baker Heart and Diabetes Institute Melbourne Australia; 6 LaTrobe University Melbourne Australia; 7 Raising Children Network Centre for Community Child Health Murdoch Children’s Research Institute Melbourne Australia; 8 Research and Knowledge Translation Parenting Research Centre Melbourne Australia; 9 Transforming Healthcare Impact Area Faculty of Medicine, Dentistry and Health Sciences The University of Melbourne Melbourne Australia; 10 Health Services and Economics Centre for Community Child Health Murdoch Children's Research Institute Melbourne Australia; 11 Curve Tomorrow Melbourne Australia; 12 Murdoch Children’s Research Institute Melbourne Australia; 13 Strategy and Growth IPC Health Melbourne Australia; 14 Sydney Local Health District Sydney Australia; 15 Sydney Medical School Faculty of Medicine and Health University of Sydney Sydney Australia; 16 Centre for Health Policy Melbourne School of Population and Global Health The University of Melbourne Melbourne Australia; 17 Centre for Community Child Health Murdoch Children's Research Institute Melbourne Australia; 18 Department of Pediatrics The University of Melbourne Melbourne Australia

**Keywords:** child mental health, consumer health information, child health services, digital health technology, family supports

## Abstract

**Background:**

Child mental health disorders are a significant Australian public health issue with high prevalence rates compounded by inequitably higher rates for those living in families with lower income, lower levels of parental education, and higher levels of unemployment. Prevention and early intervention approaches are critical to address problems early. When caregivers seek information and services to support their child’s mental health needs, they commonly use many untested online search strategies. To address this, we developed a digital Child and Family eHub (eHub) prototype through a user-centered design process involving families experiencing adversity and local service providers. The eHub provides online navigation and evidence-based information for families and aims to increase equitable access to and use of (1) information and (2) the existing primary health, mental health, and social services system to improve mental health outcomes for caregivers with children aged 0-12 years. This protocol outlines how we will evaluate the eHub.

**Objective:**

This study aims to evaluate the feasibility, acceptability, appropriateness, and preliminary impact of the eHub digital platform for caregivers of children aged 0-12 years, particularly those experiencing adversity. The evaluation will assess implementation outcomes, caregiver and child mental health outcomes, and help-seeking behaviors over a 6-month period.

**Methods:**

A prospective cohort of 270 caregivers of children aged 0-12 years will be recruited from 3 socioeconomically diverse Australian sites (Wyndham Vale in Victoria and Marrickville and Fairfield in New South Wales). Participants will be recruited through local community hubs, health and social service providers, and social media, and will enroll via the REDCap (Research Electronic Data Capture; Vanderbilt University) survey platform. A mixed methods type 3 implementation impact evaluation will be undertaken, which tests an implementation strategy while observing and gathering information on the intervention’s impact on relevant outcomes. In this protocol, implementation will be assessed as a primary outcome using Proctor’s outcomes for the implementation framework, and secondary outcomes will include caregiver access and use of the eHub and associated child and parent mental health outcomes. Data will be collected at baseline and 6 months. Quantitative data will be analyzed using descriptive statistics and regression models; repeated measures will be analyzed using generalized estimating equations. Qualitative data will be analyzed using framework analysis.

**Results:**

The study was funded in December 2021. Participant enrollment for the study began in February 2024, with participants involved in the eHub evaluation for 6 months.

**Conclusions:**

The results of this study will be instrumental in refining the intervention for future scaling to other Australian sites. This study has the potential to offer an accessible, cost-effective, and scalable digital solution to improve service navigation and mental health outcomes for children and families experiencing adversity.

**Trial Registration:**

ISRCTN Registry ISRCTN49839991; https://doi.org/10.1186/ISRCTN49839991

**International Registered Report Identifier (IRRID):**

DERR1-10.2196/72548

## Introduction

### Current Mental Health Concerns

Consistent with other international research [1,2], 1 in 7 Australian children aged 4-11 years reports mental health disorders in a year, with attention-deficit/hyperactivity disorder and anxiety being the most reported disorders [3]. Additionally, regulatory problems, such as excessive crying, sleeping, or feeding problems in infancy, are known predictors of later behavioral and mental health problems [4], with the Australian prevalence rates for 1 or more of these regulatory problems in infants between 7.3% and 25.2% [5]. The distribution of mental health disorders, however, is inequitable, with much higher rates in children and adolescents associated with lower socioeconomic status of families, lower levels of parental education, and higher levels of unemployment. Recent Australian national data identify additional mental health burden on children and their parents as a result of COVID-19 [6], with 50% of parents reporting their children as anxious [7], and even higher rates among children from families experiencing financial distress [8]. These data reinforce the urgent need to implement effective and equitable prevention and early intervention strategies to mitigate the continuing and unequal rise in child mental health issues.

### Early Intervention

Investing in innovative prevention and early intervention approaches is time critical for the following reasons: (1) despite increasing Australian government investment in mental health services (eg, over US $1.7 billion nationally in 2018-2019 to enhance community mental health services) [9], existing services are unable to meet the growing demand for children’s mental health, and there has not been a detectable reduction in the prevalence of psychological distress for youths [10]; (2) only 9%-27% of young children experiencing emotional and externalizing problems access Medicare-rebated services, with the lowest rates among families of low socioeconomic status or single parents and those with English as a second language [11]; (3) failing to intervene early costs the government (US $10.7 billion) annually [9]; and (4) there is currently strong policy commitment for mental health including the National Child Mental Health and Wellbeing Strategy [12].

To increase the efficiency of the support systems, we need to make better use of the existing (and already funded) health and social care system. In a National Child Health poll of 2000 Australian parents prior to COVID-19, only 44% reported feeling confident that they would know where to go for professional help if their child was experiencing social, emotional, or behavioral difficulties [13]. Indeed, caregivers often feel overwhelmed by the amount and complexity of information and need accessible, reliable, and relevant support to guide them through the process of accessing services relating to their child’s social, emotional, and behavioral problems [14]. Rather than looking for mental health services per se, caregivers seek practical tips, connections to a range of services, and reassurance of the best approach to take with their family and child’s mental health. These findings were recently corroborated (Honisett, PhD, unpublished data, January 2024). Low levels of access and use of community and mental health services are thought to be due to a combination of barriers including financial, cultural, transport, and health literacy barriers [15]. The process of navigating a complex community and mental health service system leads to inequities in access, missed (and costly) opportunities for early intervention, and subsequent inequities in outcomes for children.

### Caregivers’ Current Help-Seeking Behavior and Use of Information and Services

Caregivers already use a range of digital search strategies and social media to seek information and services to support their child’s health needs. These findings were recently corroborated (Honisett, PhD, unpublished data, January 2024). Common challenges for caregivers seeking online information or services are too much or not enough relevant or trusted information, lack of time to source information or services, a sense of burden felt by caregivers to find an appropriate solution, and getting “stuck” and unsure how to progress seeking appropriate support. Caregivers report that they want to find practical actions to resolve their concerns or issues, connect with existing technologies and services, and not feel judged. These findings were recently corroborated (Honisett, PhD, unpublished data, January 2024).

### Digital Solutions

Digital solutions offer great potential to provide high reach, low stigma strategies to deliver information, programs, and services [16], which can be tailored to a family’s needs. While there are many digital apps and platforms available, very few have been developed for children aged 0-12 years and their families or specifically offer service navigation support for childhood adversities or their associated mental health problems [17]. Adversities include childhood maltreatment (eg, physical, verbal, or sexual abuse) and household dysfunction (eg, parental mental illness and family substance abuse) [18] as well as broader social determinants of health—nonmedical factors that influence health outcomes, including the conditions in which people are born, grow, work, and live [19].

To address this need, our team has developed a prototype or minimum viable product (MVP) for a digital Child and Family eHub (eHub) using a user-centered design process. The process involved local service providers and caregivers experiencing adversity. The eHub aims to optimize caregiver information and local service-seeking experience and satisfaction. The user-centered design process for the eHub is outlined in previous research. The eHub connects caregivers to evidence-based information and a range of health, education, justice, and social services to support broader adversities families experience. The eHub (1) is tailored to the specific needs of caregivers with young children aged 0-12 years; (2) is designed to deliver varying levels of supported navigation based on individual need or capacity; and (3) addresses specific issues related to the social determinants of health, diversity, and reach. Improving access to information, services, and supports we hypothesize will lead to improved and more equitable mental health outcomes for children.

### Objectives

This protocol details the methods to evaluate the primary and secondary outcomes of the eHub for caregivers with children aged 0-12 years, with a specific focus on populations with low education, diverse languages, and socioeconomic deprivation. We will undertake a type 3 implementation impact evaluation as defined by Curran et al [1]. Type 3 implementation impact evaluation tests an implementation strategy while observing and gathering information on the intervention’s impact on relevant outcomes. In this protocol, implementation will be assessed as a primary outcome using Proctor’s outcomes for the implementation framework [20]. Secondary outcomes will include family information and service access and use, as well as child and caregiver mental health.

## Methods

### Study Design

A multisite type 3 mixed methods hybrid implementation or impact evaluation will be conducted [1]. The approach will assist in understanding access and use of the eHub by caregivers via the collection of users’ data analytics on the eHub platform and via a prospective cohort sample, which will provide a “deep dive” into factors affecting caregiver access and use of the eHub and associated child and caregiver mental health outcomes. Quantitative (survey) and qualitative (semistructured interview) data will be collected in parallel and integrated during data analysis and interpretation. This protocol was informed by the Standards for Reporting Implementation Studies framework [21], and where relevant, it incorporates principles from the SPIRIT (Standard Protocol Items: Recommendations for Interventional Trials) 2013 checklist.

### Site Selection

eHub sites were chosen based on populations experiencing several adversities—as defined via low socioeconomic status, cultural diversity, child vulnerability, unemployment, and low education levels. Sites in Australia include Marrickville and Fairfield in Sydney and Wyndham Vale in Melbourne (Table 1). To note, the Marrickville suburb experiences significant pockets of disadvantage among areas of advantage. These 3 sites also had an existing physical Child and Family Hub within the local service system, which integrated health and social care for children and their families.

**Table 1:** Sites involved in evaluation.

State	Site	Population details^a^				
		Socio-Economic Index for Area^b^	Australian Early Development Census—Children vulnerable in 1 domain (national comparison: 22%) (%)	Population 15+ years unemployed (national comparison: 5.1%) (%)	Population completed year 12 or equivalent (national comparison: 51.9%) (%)	Population born overseas (national comparison: 27.7%) (%)
Victoria	Wyndham Vale	972 (second quintile)	24.2	7.3	48.8	40.1
New South Wales	Marrickville	1050 (fourth quintile)	15.5	4.7	64.9	35.1
New South Wales	Fairfield	838 (first quintile)	28.6	8.7	48.2	56

^a^Percentage proportions reported for comparison as the population size varies across sites.

^b^Socio-Economic Index for Area is a group of 5 indexes that provides a relative measure of socioeconomic advantage and disadvantage for small geographic areas; 1000 is the average, lower scores indicate more disadvantage, first quintile=most disadvantaged.

### Child and Family eHub MVP

**Figure 1:**
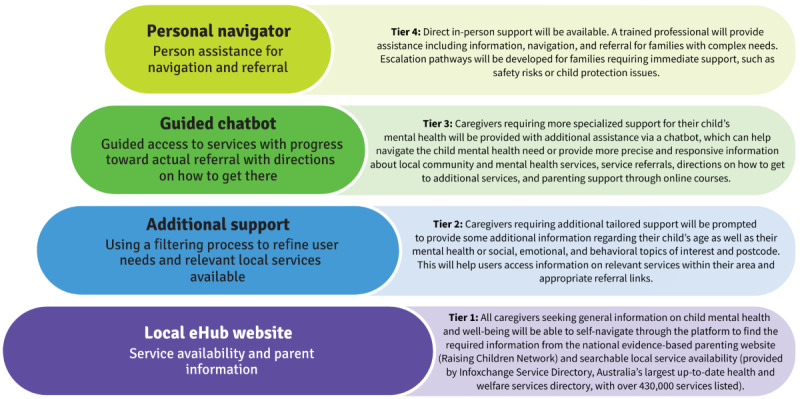
Tiers of support provided through the eHub. eHub: Child and Family eHub.

The eHub MVP was developed using user-centered design with caregivers and local service providers and aims to support and guide caregivers through a local, proportionate approach including increasing tiers of support. These tiers, outlined in Figure 1, include self-navigation for all families (tier 1) through to guided chatbot technology and finally, human navigator assistance, which has been shown to be effective [22] for those most in need (tier 4). Figure 1 also includes a brief overview of each tier of the eHub. Caregivers determine the level of support they need based on their ability to find and access information and services that suit their family needs. Each caregiver enters the eHub at tier 1 and progress to additional tiers based on their needs, for example, filtering topics or geographic areas to find appropriate information or local services (tier 2), using a chatbot to assist with defining the mental health topic most suitable to their needs (tier 3), or speaking to a personal navigator to understand the child’s or families’ specific information or service needs and assist with navigation to services (tier 4). It is anticipated that 90%-95% of eHub users will interface with tiers 1-3 based on theories of proportionate universalism [23,24], with only 5%-10% of users requiring the more intensive element of the eHub personal navigator.

### Recruitment

#### Prospective Cohort of Caregivers

**Figure 2:**
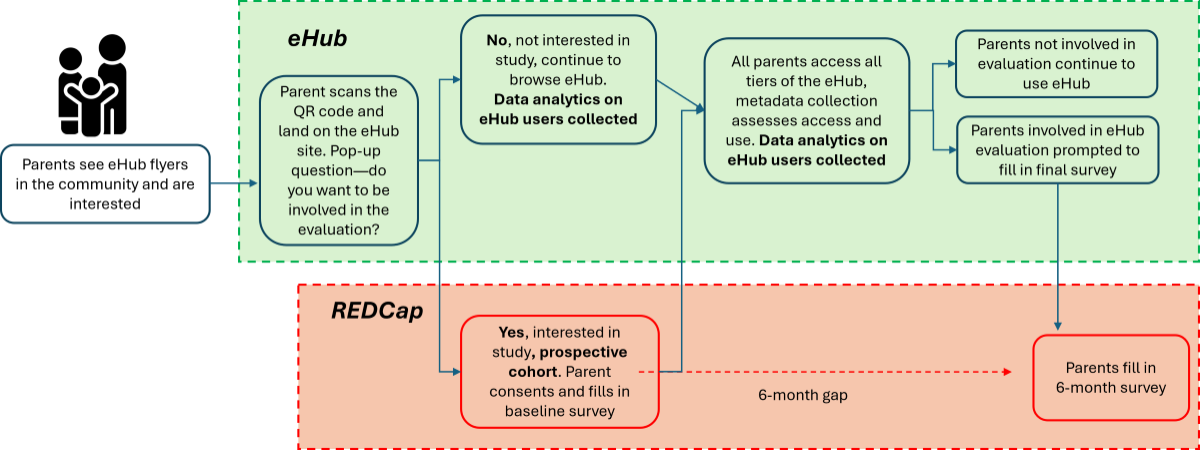
Caregiver journey for the evaluation trial. eHub: Child and Family eHub; REDCap: Research Electronic Data Capture.

The study will recruit 270 caregivers of children aged 0-12 years from the 3 identified sites (Table 1). To ensure representation of caregivers experiencing a range of adversities, the eHub will be advertised widely in the community in each site through posters and flyers with QR codes at physical primary care, community health and nongovernment organization hub sites, local community groups, schools, parent groups, playgroups, and social media to recruit caregivers with children aged 0-12 years. Interested caregivers will follow the QR code link to REDCap (Research Electronic Data Capture; Vanderbilt University)—a secure, web-based data capture platform hosted by the research team, where they can watch an informational video about the study and provide informed consent to be involved. Caregivers will then complete a baseline survey for the evaluation, including whether they meet the inclusion criteria (listed below); this will be our prospective cohort sample of caregivers. If caregivers respond “no” and would prefer not to participate in the evaluation, they can continue to use the eHub. eHub data analytics will be collected on all users of the eHub, including those involved in the evaluation and those who are not. Those not involved in the evaluation will not require consent to participate, as all eHub data analytics collected will be unidentifiable. Demographic details of participants will be collected through pop-up surveys on the eHub site. Figure 2 outlines the caregiver journey through the evaluation. Recruitment for the evaluation commenced in February 2024 and continued for each caregiver involved for 6 months.

The caregiver inclusion criteria are as follows: parents or primary caregivers with at least 1 child aged 0-12 years, noting that only 1 primary caregiver per family unit can enroll in the evaluation study; live in eligible intervention sites (Marrickville and Fairfield in New South Wales or Wyndham Vale in Victoria); can speak sufficient English to participate in the study and complete the survey, noting that although each of the study sites are culturally diverse, many residents speak English; and provide informed consent.

#### Escalation Pathways for Child or Family Concerns

To ensure appropriate clinical governance, escalation pathways were developed with each study site with support from the steering committee (1) to support caregivers if they are distressed or unsafe when calling the personal navigator related to tier 4 of the eHub and (2) to support eHub personal navigation phoneline staff.

#### Cross-Sectoral Service Providers

We will purposively recruit service provider participants who meet the inclusion criteria (listed below) within each study site to participate in semistructured interviews at 6 months from the eHub first going live. Site leads at the 3 trial sites will provide access to individual service provider contact details who may wish to take part. We will then directly approach these primary care, mental health, health, welfare, and community providers to take part, ensuring that they meet the inclusion criteria and consent to be involved in the evaluation. Many of these service providers are represented through networks that we have existing relationships within Wyndham Vale, Fairfield, and Marrickville, such as Wyndham Child and Family Alliance (Victoria) and the Local Council Vulnerable Children’s Group (New South Wales). Potential participants will be sent a personalized email of invitation to participate in qualitative interviews at the 6-month time point with information about the details of the study objectives and providing the service provider participant information sheet.

The cross-sectoral service provider inclusion criteria are as follows: adults aged 18 years or older; work within the study intervention areas—Marrickville, Fairfield, or Wyndham Vale in health, or social service located at the Marrickville Community Health Service, Karitane in Fairfield or IPC Community Health Service in Victoria; and provide verbal informed consent.

### Baseline Data Collection: Prospective Cohort Caregiver Baseline Survey

**Figure 3:**
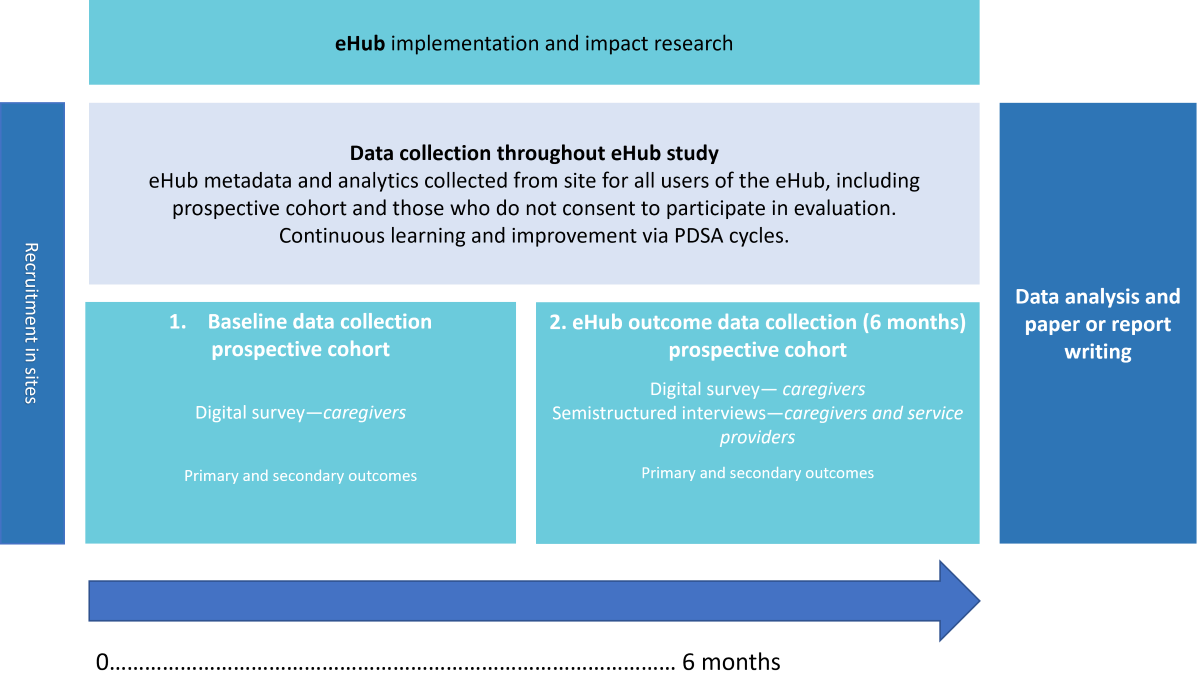
Evaluation overview. PDSA: Plan-Do-Study-Act.

For those who consent to be involved in the eHub evaluation, when accessing the eHub for the first time, caregivers’ baseline data will be collected via an online survey, which includes the research measures outlined in Table 2. This online survey will be built into a secure online portal (REDCap) [25,26] through the eHub as outlined in Figure 3. If consented families do not complete the baseline questionnaire, they will be contacted by a research assistant by phone, email, or text to complete.

**Table 2:** Study activities and timeline.

Activity	Timeline
Funding and concept development	December 2021
User-centered design and minimum viable product development	June 2022 to December 2023
Ethics approval	June 2023
Participant recruitment commenced	February 2024
6-Month evaluation period per participant	February 2024 to November 2024
Quantitative and qualitative data collection	February 2024 to February 2025
Data analysis	February 2025 to May 2025
Dissemination of findings	2026

### Data Collection Throughout the eHub Study

#### Data Analytics on eHub Users

eHub metadata and digital analytical data on participants’ average session length and frequency, page use, and tiers of service use when using the eHub, as well as satisfaction scores and responses to short online surveys and questionnaires, will be collected for any users of the eHub using Google Analytics and Hotjar (Contentsquare; a web-based feedback poll that pops up on the eHub screen during an episode of use). Additionally, brief demographic data, including postcode and country of birth of user, will be collected. Tables 3 and 4 show the primary and secondary evaluation objectives and quantitative and qualitative data collected in parallel and integrated during data analysis and interpretation.

**Table 3:** Primary evaluation outcomes and associated measures.

Primary outcomes: eHub^a^ implementation outcomes			Measure
**eHub access**			
	Reach: What is the proportion of caregivers within each site who access the eHub?	Data analytics on eHub users: Proportion of people who have accessed the eHub (numerator) among families that have children younger than 15 years of age based on Australian Bureau of Statistics reports for each site (denominator).	
	Coverage: Is the eHub reaching the intended audience of priority families (defined by residential socioeconomic disadvantage [SEIFA^b^ IRSD^c^ score in the lowest quintile or quartile?] and parental country of birth [parents born overseas?])	Data analytics on eHub users: User visits to each tier mapped against their demographic data including suburb to determine socioeconomic disadvantage, based on SEIFA scores and nomination of whether user was born overseas. This indicator measures the proportion of vulnerable families with children younger than 15 years who have accessed the eHub. Vulnerability is based on caregiver demographics—such as postcode (linked to SEIFA) and country of birth—compared to ABS caregiver data. The numerator is the number of vulnerable families who accessed the eHub, and the denominator is the total number of vulnerable 1- and 2-parent families with children younger than 15 years.	
**eHub use**			
	Engagement: Are families engaging with the eHub?	Data analytics on eHub users: Do caregivers passively use the eHub? Page hits, links opened to information, services within each tier of the eHub, and actively use the eHub, that is, searches undertaken through tiers 2 and 3 and clicking through to tier 4 support. eHub user data will include: Length of time on site or session length Visits or time per critical feature Interactions with filters Number of engaged sessions and engagement rate Engaged sessions per user or IP address Average engagement time Views per session or user	
	Acceptability: Do caregivers view the eHub as satisfactory?	Data analytics on eHub users: Pop-up surveys on satisfaction, Did you find what you were looking for? Or Was this useful? (Thumbs up or down or smiley or sad face) Survey (6 months): Satisfaction with content, delivery, level of support provided, credibility.	
	Adoption: To what extent can caregivers find and use information, services, and supports on the eHub?	Prospective cohort survey and interview (6 months): Did caregivers find the information provided useful for their needs? Were caregivers able to find an appropriate service or information to support their or their child’s needs? Did caregivers use or intend to use the strategies provided in the information? Changes to caregivers’ behavior (eg, help-seeking and implementing new strategies)	
	Appropriateness: Do caregivers perceive the eHub as useful and relevant? Did the caregiver feel reassured or supported by the information or services provided?	Prospective cohort survey (6 months): Perceived fit, relevance, compatibility, suitability, usefulness, practicability.	
	Barriers and enablers: Accessing and using information, services, and supports on eHub?	Prospective cohort caregiver and service provider qualitative interviews (6 months).	
**Cost**			
	Cost of providing the eHub, intersectoral service use, and effectiveness of eHub	Prospective cohort survey (6 months): Costs of implementation and national scaling of the model will be estimated (using study protocols, budgets, and population of children). The costs associated with caregiver-reported intersectoral service use will be estimated. Cost-effectiveness estimated based on the number of families engaged and those with unmet needs being met.	
**Demographics**			
	Postcode, single or couple parent, country of birth, language spoken at home, education status, number of adversities	Prospective cohort survey (baseline): The population cohort—eHub metadata will provide demographic characteristics of caregivers who access the eHub, including postcode to link SEIFA and parents’ place of birth compared with caregiver data from ABS.	

^a^eHub: Child and Family eHub.

^b^SEIFA: Socio-Economic Index for Area.

^c^IRSD: Index of Relative Socio-Economic Disadvantage.

^d^ABS: Australian Bureau of Statistics.

**Table 4:** Secondary evaluation outcomes and associated measures.

Secondary outcomes: impact		Measure
**Information and service access and use**		
	Service access and use: Did the eHub assist caregivers with finding and connecting with relevant services including those relevant to social determinants of health and adversity? If not, why?	Prospective cohort survey (baseline and 6 months): Using the general [27] and actual [28] help-seeking questionnaires: Intention to seek help Actual help-seeking
	Information access and use: Did caregivers find the information needed? If not, why? Did accessing information change the caregiver’s behavior?	Prospective cohort survey (6 months): Did you find the information you needed? If not, why?
**Caregiver mental health**		
	Caregiver distress: Is eHub exposure associated with improvements in caregiver levels of distress?	Prospective cohort survey (baseline and 6 months): Kessler Psychological Distress Scale (K10) [29]
	Caregiver stress: Is eHub exposure associated with reduced caregiver stress related to (1) access to services and (2) their child’s mental health?	Prospective cohort survey (baseline and 6 months): Study designed Likert scale of stress
**Child mental health**		
	Child mental health needs met: Did the caregivers’ child have unmet mental health care needs? If yes, were they met by the eHub resources?	Prospective cohort survey (baseline and 6 months): Unmet child mental health need [30]
	Child mental health symptoms: Is eHub exposure associated with improvements in parent-reported child mental health symptoms?	Prospective cohort survey Child aged 2 months to 2 years—Survey (baseline and 6 months) of Well-Being of Young Children [31] Child aged 2-12 years—Strengths and Difficulties Questionnaire [32]

#### Continuous Learning and Improvement of the eHub Throughout Evaluation

Members from each evaluation site will form a project team alongside researchers to identify and respond to barriers for caregivers in accessing and engaging with the eHub. Using implementation science activities, the team will conduct 2-3 short Plan-Do-Study-Act quality improvement cycles [33] over the 6 months of implementation, including review of caregiver satisfaction with the navigation processes and content and whether caregivers are progressing to higher tiers of support. These short learning cycles will allow agile minor improvements of the eHub.

### eHub Outcome Data Collection (6 Months)

#### Prospective Cohort Caregiver Outcome Surveys (6 Months)

Caregivers involved in the evaluation will be sent an email or SMS text message invitation to complete an online survey at 6 months after baseline to measure outcomes on access and use of health services, accessibility, and overall experience of using the eHub. Secondary outcomes will also be measured via the caregiver outcome survey. All measures and associated data are outlined in Table 3. Surveys will be collected in REDCap, a secure online portal. Research assistants will follow-up those caregivers who do not complete the caregiver outcome survey via phone, text, or email.

#### Prospective Cohort Caregiver and Cross-Sectoral Service Provider Semistructured Interviews

Caregivers (n=15-20 in total) and intersectoral service providers (n=15-20 in total) from the evaluation sites will be invited to participate in individual semistructured interviews at 6 months after eHub implementation. Interviews will be conducted by web-conferencing platform (Zoom; Zoom Video Communications). Participants will be invited to provide their perspectives on barriers and enablers of using the eHub, such as were caregivers’ needs satisfied by using the eHub? Was the eHub able to support their unmet social needs? Did caregivers use or intend to use strategies provided in information sourced on the eHub? And were there changes to caregivers’ behavior (eg, help-seeking and implementing new strategies)? (see Multimedia Appendix 1 for interview guide). Participants will be invited to expand on any points they feel are important, and the interviewer may ask follow-up questions to elicit further details. The research assistant will be trained and experienced in qualitative methods; the interviews are expected to last between 20 and 30 minutes.

### Statistical Analysis

#### Sample Size Estimation for Impact Evaluation

We will recruit a total of 270 caregivers to give the study 90% power to detect an 11% increase in the proportion of caregivers accessing services via the eHub, that is, from 9% to 20% based on previous research [34], with a hypothetical 5% increase without the intervention. This allows for a 10% loss to follow-up.

#### Statistical Methods for All Quantitative Outcomes

We will describe the characteristics of the caregivers and children using the mean (SD) and median (IQR) for continuous data (based on data distribution) and proportions for categorical data, at each time of measurement. To evaluate changes in key outcomes over time (baseline and 6 months), we will analyze the prospective cohort survey data using logistic and linear regression models for binary and continuous outcomes, respectively, specified in Table 3. Binary outcomes will be reported as odds ratios (as change in proportions) with 95% CIs; continuous outcomes will be reported as mean differences (as change in means) with 95% CIs. We will conduct a generalized estimating equation approach to account for repeated measures (ie, baseline and 6 months) for each participant. We will undertake stratified analyses by service site to explore whether outcomes vary by site. In consideration of the change in participants’ characteristics between baseline and 6-month surveys due to loss to follow-up, we will control for family socioeconomic characteristics. Missing data will be handled using appropriate statistical techniques (such as multiple imputation for missing at random). We will describe the proportions of measures derived from data analytics from eHub users (specified in Table 3). We will conduct all analyses using STATA (version 16) or SAS (SAS Institute) statistical software packages.

#### Qualitative Data Analysis: Caregiver and Intersectoral Service Provider Interviews

Experienced research assistants at each site will use deductive and inductive analysis of stakeholder interviews using framework analysis. Framework analysis is suitable for this applied study because the technique is not aligned with any specific epistemological stance and places the research questions at the forefront of the analysis [35]. Interviews will be transcribed, and NVivo (version 14; Lumivero) will be used to code data from interviews. The first 10% of interviews at each site will be double-coded by research assistants, and any issues will be resolved prior to finalizing coding for all interview participants.

#### Health Economic Analysis

Using study protocols and budgets, the cost of implementing the eHub model will be estimated. This will include fixed or upfront costs of building the eHub as well as variable ongoing costs of maintaining and staffing. The cost of caregiver-reported intersectoral service use will be estimated. Cost effectiveness will be calculated as a cost per additional child or caregiver engaged with the program and a cost per additional child with unmet needs who accesses the eHub model. The cost of a national roll-out of the eHub model will be estimated based on the number of children serviced by the study versus the whole Australian population of children. An estimation will be made on the number of eHub local modifications needed nationally.

### Ethical Considerations

This study received ethics approval from the Royal Children’s Hospital Human Research Ethics Committee (HREC #84970). All participants will provide informed consent prior to enrollment. Caregivers will access study information and consent materials via a secure online platform (REDCap) and will have the opportunity to ask questions before providing electronic consent. Participation is voluntary, and individuals may withdraw at any time without consequence. All data collected during the study will be deidentified prior to analysis. Participant information will be stored on secure servers in accordance with institutional data security protocols to protect privacy and confidentiality. Only approved members of the research team will have access to identifiable information, which will be stored separately from deidentified datasets. Participants will be reimbursed with a US $20 gift voucher for their time and contribution to the study.

## Results

This study received funding in December 2021, followed by user-centered design of the eHub MVP with families and local service providers. The study was registered with the ISRCTN Registry in June 2023. Following ethics approval in June 2023, participant enrollment and data collection for the study began in February 2024, with participants involved in the eHub evaluation for 6 months. Analysis of data was conducted from February 2025 to May 2025. Table 2 outlines the study timelines across major phases.

## Discussion

This study anticipates that the eHub will be a feasible, acceptable, and appropriate digital solution for caregivers of children aged 0-12 years, particularly those experiencing adversity. We hypothesize that implementation of the eHub will result in increased access to and engagement with relevant health and social services, improved caregiver satisfaction with the information-seeking process, and reductions in caregiver distress and child mental health concerns.

Despite significant increases in mental health needs, families with young children (aged 0-12 years), especially from priority groups, are still not accessing services and supports early, and they often present after complexities and comorbidities have set in and experience a significant emotional burden of seeking help [14]. Lack of timely access to supports and services is further compounded by the poorly coordinated and fragmented Australian child and family health and social care service systems that families find difficult to navigate. While there are some digital applications available for practitioners to help families support their children’s developmental needs in the preschool period, including coordinating wraparound social care [36,37], none of the available tools focus on families of children aged 0 to 12 years with mental health concerns, while also incorporating support for broader social determinants of health and mental health, such as housing, finances, food insecurity, employment, and legal issues.

The eHub provides online navigation and evidence-based information for families and aims to increase equitable access to and use of (1) information and (2) the existing primary health, mental health, and social services system to improve mental health outcomes for caregivers with children aged 0-12 years. Whether these digital applications can be feasibly implemented and adopted, especially within diverse and limited-income populations within Australia, remains largely unknown.

This protocol outlines a mixed methods implementation-impact evaluation that uses Proctor’s outcomes framework [20] to assess the reach, engagement, adoption, appropriateness, and acceptability of the eHub. Quantitative and qualitative data will be collected in parallel to examine user access patterns, satisfaction, and changes in help-seeking behaviors and caregiver or child outcomes. The majority of caregivers are expected to engage with the lower tiers of the eHub (tiers 1-3), consistent with a proportionate universalism approach, with a smaller proportion using tier 4 (human navigation support).

In this protocol, we outline the methodology to assess the uptake, usability, acceptability, appropriateness, and cost of the eHub, noting a complementary quality improvement approach to improve as implemented [1]. This study builds on the MVP that has already been developed, and it will validate the design by testing the eHub, optimizing its existing functions, and informing future features.

While several digital tools exist, few have been specifically developed for children aged 0-12 years or explicitly address the intersection of mental health and social adversity [38,39]. Internationally, digital platforms to support child and family mental health have emerged in various forms; however, most are targeted toward adolescents or focus solely on mental health content without integration of social care navigation. For example, programs like the United Kingdom’s “Kooth” platform [40] or the US-based “MyStrength” [38] target older populations and are not co-designed with caregivers of young children. In contrast, the eHub is uniquely tailored for caregivers of children aged 0-12 years, combining access to both health and social service information with a user-centered design approach involving families experiencing adversity. This locally grounded, system-oriented model may offer transferable insights for international contexts facing similar challenges related to service fragmentation. The eHub directly addresses the literature gap by integrating co-designed content with tiered navigation supports, helping caregivers overcome common barriers to service access such as health literacy limitations, time constraints, and stigma.

Given the current gap in the existing “market” for this type of digital service, it will be essential to consider how best to learn from the evaluation to enhance its replicability to other sites. Furthermore, this study will provide insights into potential mechanisms underlying the intervention’s impact; shed light on the effectiveness, or ineffectiveness, of its various components; and offer possible explanations that will, again, be essential for replication.

Strengths of the study include its multisite design across 3 diverse communities, the use of real-world user data and embedded quality improvement processes, and the integration of caregiver and provider perspectives through semistructured interviews. One potential limitation of this study is the relatively short 6-month evaluation period, which may not capture long-term impacts on mental health outcomes or sustained service use. However, this time frame may be appropriate, given that digital applications are often designed for targeted, time-limited use, with peak engagement typically occurring during periods of greatest need or relevance to users. Another limitation is the inclusion criterion requiring sufficient English proficiency to participate in the study and complete the survey. Although each study site is culturally diverse and many residents speak English, this focus may limit the generalizability of findings to caregivers from non-English–speaking backgrounds.

Despite these limitations, this study will provide critical evidence on whether the eHub is effective in meeting the needs of priority populations and addresses inequitable access and uptake of support and services for families who are experiencing a range of adversities. Future directions may include testing longer-term outcomes of eHub use, examining cost-effectiveness over time, and adapting the platform for broader populations, including those speaking languages other than English. Results from this study may also inform the development of an implementation guide to support scale-up to other Australian sites.

Dissemination of the study findings will include publication in a peer-reviewed journal paper. On completion of the trial, and after publication of the paper, results will be presented to relevant state and federal government contacts and presented at relevant domestic and international conferences. Feedback will also be shared with participating community partners and made accessible through the eHub platform to ensure that knowledge is translated back to service users and providers.

In summary, the results of this study will provide much-needed data and insight into the wider sustainable implementation of the eHub to facilitate equitable access to relevant evidence-based information and services to support child and family mental health. In addition, this project has the potential to increase our understanding of how digital solutions can improve navigation to information and services for a variety of health and social issues in a cost-efficient manner at a national level.

## Appendix

Multimedia Appendix 1 Caregiver interview guide.

Multimedia Appendix 2 Peer review report from the National Health and Medical Research Council, Australia, and The Royal Children’s Hospital Melbourne.

## Abbreviations

eHub: Child and Family eHub
MVP: minimum viable product
REDCap: Research Electronic Data Capture
SPIRIT: Standard Protocol Items: Recommendations for Interventional Trials
