# Supplementary material for: A Novel Digital Platform to Support Child and Family Mental Health in Australia (Child and Family eHub): Protocol for a Mixed Methods Evaluation
Source: JMIR Res Protoc. 2025 Nov 13;14:e72548. doi: 10.2196/72548 (PMC12661229; doi:10.2196/72548)
Supplement: Multimedia Appendix 1 [file resprot_v14i1e72548_app1.docx]

**Introduction**

Thanks for continuing to be part of the Child and Family eHub study.

As you know many families struggle with knowing what services are available and appropriate for their children’s health and development concerns. The Child and Family eHub offers a digital “front door” to existing services and programs that support the health and social care needs of children and their families.

Today we are going to ask you about your thoughts on using the Child and Family eHub. This will help us better understand if families are finding it useful and how we can improve the eHub going forward.

| **Key theme** | **Key issues to draw out** | **Interview questions** |
| --- | --- | --- |
| **Adoption and uptake (The intention, initial decision, or action to try or employ an innovation or evidence-based practice)** |  | Q: In the past 6 – 8 months how many times have you been on the Child and Family eHub site? If you don’t remember, please give us your best guess? |
|  | - The intention, initial decision of engaging | Q: What prompted you to use the eHub?  Q: Were there particular issues that you used the eHub for? If so, what were they?  Q: Were there particular supports that you used the eHub for – information, services, online parenting programs, connections to other parents?  Q: Were there supports that were not available on the eHub site that you hoped to find? |
|  | - If connected with services, was it helpful for your child? (See CRE questions) - If not connected with services, why not (See CRE questions which consider wait lists, transport, cost etc.) | Q: Focusing on services, did the eHub assist you with finding and connecting with relevant services?  If yes, have you used any of those services used in the last 4 months? How many times did you access these services?  Q: Were these services helpful for your child?  If no, what stopped you from connecting with services?  Did you face any issues like wait lists, transportation problems, or costs? |
| **Appropriateness (the perceived fit, relevance or compatibility of the innovation or evidence-based practice for a given practice setting, provider or consumer: and/or perceived fit of the innovation to address a particular issue or problem)** | - Did caregivers find *information* provided useful for their needs? - Were caregivers able to find an appropriate *service or information* to support their/their child’s need? - Did caregivers use or intend to use strategies provided in information? - Changes to caregivers’ behaviour (e.g., help-seeking, implementing new strategies) | Q: Were you able to find an appropriate information to support you and/or your child’s needs?  If yes: If yes, did you use or do you intend to use any strategies provided in information found on the eHub?  If no: What was your experience?  Q: Did you call the eHub phoneline support number?  If yes, did you find it useful to speak to an eHub navigator?  Was the information and/or services recommended by the eHub navigator useful to your child and/or your needs?  If no, was there any particular reason why you did not call the phoneline support number?  Q: Did you find the eHub relevant & useful?  If yes: Can you share what you liked and what you found useful on the eHub site?  If no: What changes would make the eHub more useful for you and your family? |
|  | - Did the caregiver feel reassured/supported by the information or services provided? | Q: Overall, did you feel reassured/supported by the information and/or services provided on the eHub? |
| **Acceptability (Perception that the innovation is agreeable, palatable, or satisfactory)** | - Did you find it satisfactory… | Q: How satisfied are you with the eHub?  How could the eHub be improved for you and your family? |
| **Barriers/enablers** | - What were the barriers and enablers to accessing and using information/services on the eHub (Semi-structured interviews) | Q: Did you encounter any challenges when trying to access information or services on the eHub?  Q: Was there anything in particular that made it easier for you to access information or services on the eHub? |
| **Wrap Up** |  | Q: Do you have any final feedback on your experience using the eHub and how we could improve the eHub going forward? |

Thanks so much for your time today, we really appreciate all of the feedback you have provided. We will send you a list of support numbers in case you found anything distressing from today’s interview and would like additional support (send a copy of Appendix 15).
