# Supplementary material for: A Novel Digital Platform to Support Child and Family Mental Health in Australia (Child and Family eHub): Protocol for a Mixed Methods Evaluation
Source: JMIR Res Protoc. 2025 Nov 13;14:e72548. doi: 10.2196/72548 (PMC12661229; doi:10.2196/72548)
Supplement: Multimedia Appendix 2 [file resprot_v14i1e72548_app2.pdf]

The Royal Children's Hospital Melbourne

Pre-Submission (Peer) Review Proforma

The purpose of the review is to identify areas for improvement which will ensure the project is scientifically valid. Please also see Pre-submission (Peer) Reviewer Process for more information.

**PROJECT TITLE**	Evaluation of a digital eHub to improve access and utilisation of existing primary health, mental health, and social services 84970
**Version number & date of Protocol under review**	Version 1

**PRINCIPAL INVESTIGATOR**	Prof Sharon Goldfeld
**Position Title**	Director | The Centre for Community Child Health<br><br>Theme Director Population Health | Co-Group Leader Policy and Equity, Murdoch Children's Research Institute<br>Professor | Department of Paediatrics, The University of Melbourne
**Department / Group**	Population Health
**Institution**	Murdoch Childrens Research Institute

**PEER REVIEWER NAME**	Dr Simone Darling
**Position Title**	Senior Program Manager and Research Fellow
**Department / Group**	Mental Health Research & Policy, Centre for Community Child Health
**Institution**	Murdoch Children's Research Institute

PEER REVIEWER DECLARATION:

- I agree to maintain confidentiality of all matters and documents regarding this project; and
- I am independent of this project; and

☐ I agree that I have no potential conflicts of interest in reviewing this research protocol; **OR**

☐ I declare I have the following potential conflicts of interest:

Please disclose any actual or potential conflict of interest in the research being reviewed, including any:

a) Personal involvement or participation in the research

b) Financial or other interest or affiliation, or

c) *Involvement in competing research*

Please explain any 'No' response and also record any comments regarding required changes or suggestions which could improve the project in the relevant section on the last page of this form.

**CRITERIA:** *Using the right column please indicate if each criteria has been addressed, in your opinion*		**YES NO N/A**
**Project details:** Has all appropriate information been included? (Investigator details and project title, protocol version number and date)		Yes
**Research question:** Is there a clearly and precisely defined, answerable question? Is there a clear aim or objective?		Yes
**Background:** Is the research question an important one? Does the background information provided give a good rationale for why the project is being done? Is the study useful to clinical practice? Is there a real problem/ knowledge gap that needs filling?		Yes
**Plan of Investigation:**		
1	**Design:** is the design appropriate to the aim? Will the study address the question being asked and is it likely to produce an answer?	Yes
2	**Bias and confounding**: Has the study been designed to minimise the risk of bias? Have the investigators adequately accounted for the influence of potential confounders?	Yes
3	**Randomisation and Blinding:** Where applicable, is enough detail provided on exactly how randomisation and blinding will be achieved, including who is responsible?	NA
4	**Sampling issues:** Will the proposed study group be large enough to provide sufficient statistical precision or power, where appropriate? Is there a reasonable justification for the proposed sample size? Will the sample collected be reasonably representative of the population in question?	Yes
5	**Feasibility:** Is there sufficient evidence to indicate that it will be possible to obtain the numbers required for the study? Is the study feasible in terms of funds, time and other resources?	Yes
6	**Participants:** Are the criteria for eligibility clear and justified? Have the methods used to identify, approach, recruit and consent participants been clearly and completely described?	Yes
7	**Intervention or exposure:** Is the intervention or exposure factor clearly described in adequate detail, where appropriate? If the intervention is a drug, are details of dose, delivery, preparation, handling and compliance provided?	Yes
8	**Procedure plan:** Has an appropriate plan of the study been detailed? Is the estimated duration of the project stated and appropriate? Is it clear how a participant will progress through treatments, procedures, assessments and visits, where applicable?	Yes
9	**Outcome measures:** Are these appropriate and achievable? Are definitions sufficiently detailed? Is the relevant data being collected on the proposed outcomes?	Yes
10	**Adverse Events:** Is there an appropriate plan for detecting, managing, recording and reporting defined adverse events, where applicable?	Yes

11	**Data collection:** are the proposed data collection tools and data management systems appropriate for the project?	Yes
12	**Analysis:** is there an adequate indication of what analysis will be done on outcome measures to answer the research question? Are the proposed analyses appropriate?	Yes
**Project management:** have adequate arrangements been specified for conduct and oversight?		Yes
**Expertise:** Does the research team include (or have access to) all the necessary expertise for the project?		Yes
**Ethical issues:** Have any potential ethical issues been addressed? Are risks to participants minimised?		Yes

Each question, comment, suggestion or requirement should be separately bulleted.
Where applicable please reference the section and page number.

**General Comments** (Remarks that the investigator does not need to respond to)
▪ This is a well-designed study that will significantly contribute to the field. All components of the protocol are included and comprehensive.

**Required Changes** (Points that the investigator must address by either making the required change, or producing a cogent argument against the change)
▪ NA

**Suggested Changes** (Points that the reviewer thinks may improve the project. They are not of such importance that they would render the project scientifically invalid/unethical if the investigator did not address the issues)
Consider the following:<br>• Provide more context around stage 1 and stages 2 and 3. Make it clear that this application is related to but separate to stage 1.<br>▪ Updating dates throughout the document as they do not appear to align - sometimes refers to timeline of all stages, sometimes just stages 2 and 3<br>▪ Updating section 2.5, it seems to be incomplete<br>▪ Aligning the aims in section 1 and 3.4 – they seem slightly out of alignment<br>▪ Ensuring there is a research collaboration agreement in place to cover transfer of data between organisations, confidentiality and IP<br>▪ detailing how a primary care giver is defined, and if two caregivers from one family can participate<br>▪ adding perceived coercion as a risk<br>▪ including the list of recommended resources for distressed participants as an appendix<br>▪ adding details about delivering focus groups/interviews face to face (as mentioned in 9.2)<br>▪ who will do the transcription of the qualitative data. If it’s a third party provider, ensure a service level agreement is in place and they are an approved supplier via MCRI legal<br>▪ minor typos throughout

**Pre-submission review outcome:** (reviewer to circle)	
**A**	**No changes required:** take the study forward to submission.
**B**	**Changes suggested:** at the discretion of the investigator; take the study forward to submission.
**C**	**Changes required:** decision about acceptability of subsequent changes at the discretion of the HREC representative. A peer reviewer does not need to review the amended protocol prior to submission.
**D**	**Changes and further peer-review required:** decision about acceptability of the subsequent changes at the discretion of the reviewer following a pre-submission peer review of the amended protocol. An additional review & proforma should be completed to document the review of the amended protocol.

**Peer Reviewer Signature**	[signature]	**Date**	16/3/23
